# Application of dual-trajectory screws in revision surgery for lumbar adjacent segment disease: a finite element study

**DOI:** 10.1186/s13018-022-03317-9

**Published:** 2022-09-24

**Authors:** Jincheng Wu, Dongmei Yang, Ye Han, Hanpeng Xu, Wangqiang Wen, Haoxiang Xu, Kepeng Li, Yong Liu, Jun Miao

**Affiliations:** 1 Department of Spine Surgery, Tianjin Hospital, Tianjin University, Jiefangnanlu 406, Hexi District, Tianjin, 300210 China; 2 Southern Medical University, Guangzhou City, Guangdong China; 3 The Affiliated Hospital of Hebei University, Baoding City, Hebei China; 4 The First Affiliated Hospital of Hainan Medical University, Haikou City, Hainan China; 5 The Second People’s Hospital of Hefei, Hefei, Anhui China; 6 Second Central Hospital of Baoding, Zhuozhou City, Hebei China; 7 Pingyao County Traditional Chinese Medicine Hospital, Jinzhong City, Shanxi China

**Keywords:** Revision surgery of ASDz, Posterior lumbar interbody fusion (PLIF), Dual-trajectory screws, Cortical bone trajectory (CBT), Finite element analysis

## Abstract

**Background:**

Advancements in medicine and the popularity of lumbar fusion surgery have made lumbar adjacent segment disease (ASDz) increasingly common, but there is no mature plan for guiding its surgical treatment. Therefore, in this study, four different finite element (FE) ASDz models were designed and their biomechanical characteristics were analysed to provide a theoretical basis for clinical workers to choose the most appropriate revision scheme for ASDz.

**Methods:**

According to whether internal fixation was retained, different FE models were created to simulate ASDz revision surgery, and flexion, extension, axial rotation and lateral bending were simulated by loading. The biomechanical characteristics of the adjacent segments of the intervertebral disc and the internal fixation system and the range of motion (ROM) of the lumbar vertebrae were analysed.

**Results:**

The difference in the ROM of the fixed segment between FE models that did or did not retain the original internal fixation was less than 0.1°, and the difference was not significant. However, the stress of the screw–rod system when the original internal fixation was retained and prolonged fixation was performed with dual-trajectory screws was less than that when the original internal fixation was removed and prolonged fixation was performed with a long bar. Especially in axial rotation, the difference between models A and B is the largest, and the difference in peak stress reached 30 MPa. However, for the ASDz revision surgery segment, the endplate stress between the two models was the lowest, and the intradiscal pressure (IDP) of the adjacent segment was not significantly different between different models.

**Conclusion:**

Although ASDz revision surgery by retaining the original internal fixation and prolonging fixation with dual-trajectory screws led to an increase in stress in the fusion segment endplate, it provides stability similar to ASDz revision surgery by removing the original internal fixation and prolonging fixation with a long bar and does not lead to a significant change in the IDP of the adjacent segment while avoiding a greater risk of rod fracture.

## Introduction

Societal advances and the growth of the ageing population have led to an increasing recognition of lumbar degenerative diseases among spinal diseases, for which lumbar fusion plays an important role in the surgical treatment, enhancing the stability of the spine with a clinically confirmed therapeutic effect [1]. The traditional trajectory (TT) screw is the most classic surgical tool used in lumbar fusion; it can provide strong internal fixation, promote bone graft fusion and ensure the stability of the spine. However, advances in medical technology in recent years have helped promote the use of cortical bone trajectory (CBT) screws, which, compared with TT screws, cause only mild denudation of the soft tissue and less nerve and vascular damage and have a greater anti-pullout force [2]. However, due to the intraoperative resection of the lamina and intervertebral disc, injury of the facet joint and fusion of long segments, the range of motion (ROM) and stress of adjacent segments are increased, thus accelerating the degeneration of adjacent segments [3–5]. As a result, a series of resulting clinical symptoms affects the patients’ quality of life. According to previous biomechanical and clinical studies, the incidence of lumbar adjacent segment degeneration (ASDeg) and adjacent segment disease (ASDz) ranges from 17 to 84% and 1% to 43%, respectively, while the incidence of ASDz requiring a second surgical procedure within 5 years was 16.5% but as high as 36.1% within 10 years [6–8]. ASDz can cause severe back pain, nerve root symptoms or neurogenic intermittent claudication, which can affect the patient's quality of life [9]. Additionally, the difference in the possibility of postoperative ASDz and the need for surgery between different types of lumbar fusion was not significant [10, 11]. As there is no unified guidance for the surgical treatment of ASDz, for clinical workers, there is no high-quality evidence to identify which surgical method is superior. At present, the traditional strategy for the treatment of ASDz is to prolong the previous screw–rod system, but this will lead to a longer operation time and increase the difficulty of the operation and the possibility of postoperative complications [12–14]. In the selection of surgical method for ASDz revision surgery, some clinical workers choose to retain the original internal fixation and prolong fixation with dual-trajectory screws. This operation method retains the original internal fixation instruments, avoids re-incision of the original scar tissue, has little soft tissue exposure and reduces the operation time and intraoperative bleeding. Previous studies have reported that the pedicle of 50% of patients can achieve dual-trajectory screws [15]. This provides us with another choice, but there is a lack of long-term follow-up to evaluate the effect of the operation.

Even though some cases have reported the use of dual-trajectory screws in ASDz revision surgery, as far as we know, the specific mechanical characteristics have not been experimentally investigated. The finite element method can well simulate the motion state of vertebral body, so that it can well analyse the biomechanical characteristics of each vertebral body structure and internal fixtures, and tell us more intuitively the mechanical changes in human body when the dual-trajectory screws technology is applied. Therefore, this study intended to use the FE models to evaluate the biomechanical characteristics of different ASDz revision surgery. The mechanical characteristics of different surgical methods were compared by comparing the related data of different surgical models, such as the ROM of fusion segments, the VMS of the screw–rod system, the VMS at the interface between the cage and the L3 upper endplate, and the IDP of the adjacent intervertebral disc.

## Materials and methods

### Intact FE model

Data of the L1-S lumbar spine FE model were collected from a healthy adult male volunteer (24 years old, weight 67 kg, height 173 cm). The volunteer had no previous history of trauma or fracture. Any spinal diseases were excluded by clinical imaging examination to establish a normal intact FE model. The volunteer was recruited by the Spinal Surgery Department of Tianjin Hospital and signed informed consent forms in accordance with the relevant regulations, which were submitted to the Ethics Committee for approval. A 64-slice spiral computed tomography scanner (GE, Siemens Sensation 16 Slice, Germany) was used to obtain the tomographic image data of the L1-S1 vertebrae with a 0.625-mm interslice interval in DICOM format. The image data were imported into Mimics 20.0 (Materialise, Belgium) to create a 3D surface model of the L1-S1 vertebrae and then into 3-Matic 12.0 software (Materialise) in STL format to perform wrapping and smoothing operations, remove excess triangular patches and initially establish the structure of intervertebral disc and nucleus pulposus for exporting into Geomagic Studio 12.0 (Geomagic, Cary, NC, USA). After smoothing and accurate surface processing, the model was imported into HyperMesh 2017 (Altair Engineering, Troy, MI, USA) for mesh division and ligament construction and finally into Abaqus 2019 (Simulia, Johnston, RI, USA) for model assembly, material property definition and finite element analysis.

**Fig. 1:**
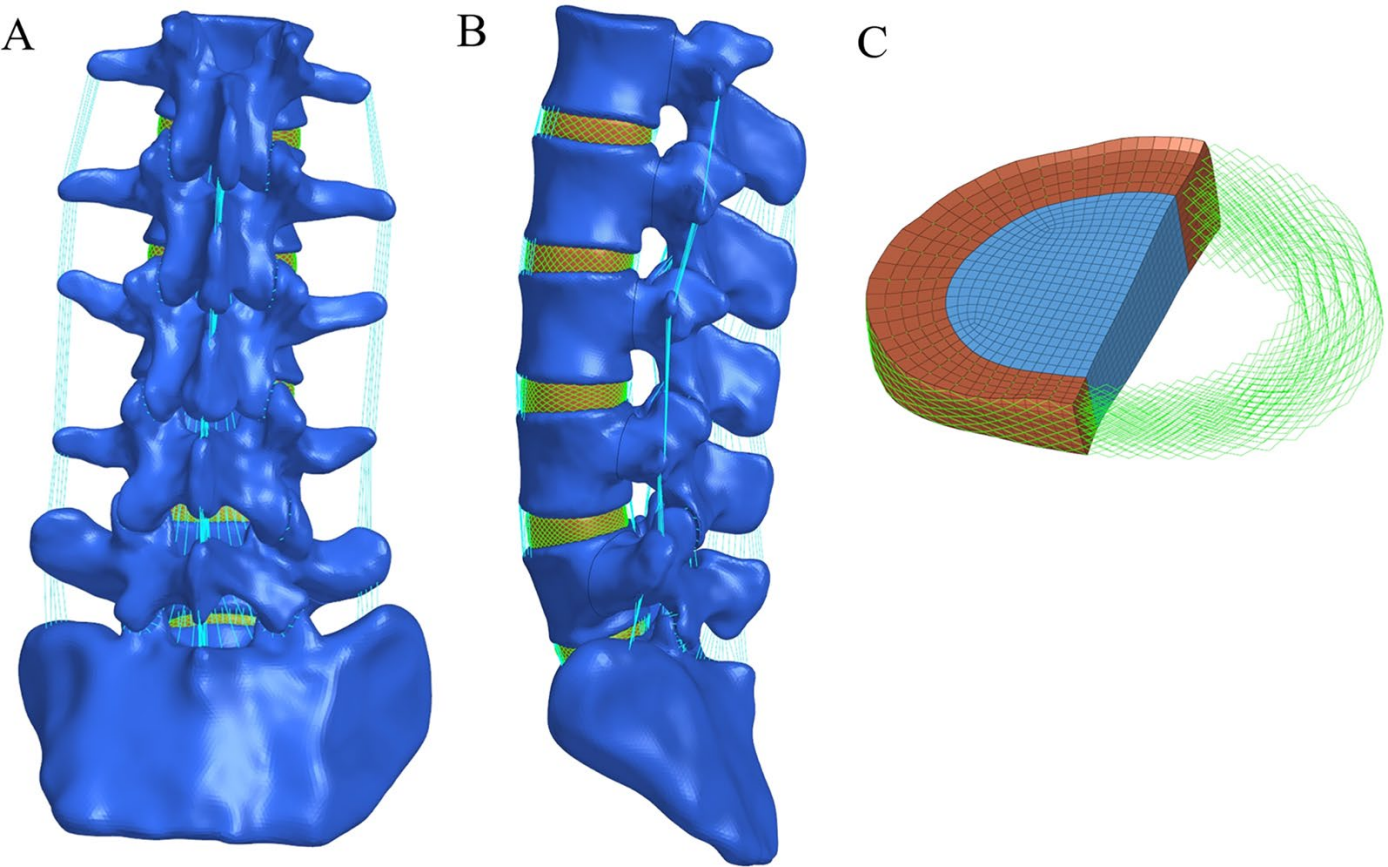
FE models of the intact L1-S lumbar spine in the current study. **A** Posterior view, **B** side view, **C** intact intervertebral disc

As shown in Fig. 1, a three-dimensional FE model of the normal L1-S1 lumbar vertebrae was constructed. The intervertebral disc is composed of the annulus ground substance, nucleus pulposus, annulus fibres and cartilaginous endplate, of which the nucleus pulposus accounts for 43% of the total disc [16]. Ligaments were simulated by using a tension-only truss element [17], and five layers of fibres were constructed from inside to outside and embedded into the annulus ground substance at an inclination of ± 30°. The elastic strength of the annulus fibres increased proportionally from the innermost (360 MPa) to the outermost fibres (550 MPa) [18–20]. Each vertebra was divided into cortical, cancellous and posterior bone structures, in which the cortical bone, cartilaginous endplate and cartilage layer of the facet join were simulated by shell elements with thicknesses of 1 mm, 0.5 mm and 0.2 mm, respectively [21, 22]. Facet contact surfaces were defined as surface-to-surface contacts with a friction coefficient of 0.1 [23]. The mesh convergence of the intact L1-S model was tested, which contained 580,440 elements and 155,100 nodes. The material properties were defined according to the previously reported literature [18, 24, 25], as shown in Table 1.

**Table 1:** Material properties used by the finite element model

Component	Young’s modulus (MPa)	Poisson’s ratio	Cross-sectional area (mm^2^)
*Vertebra*			
Cortical bone	12,000	0.3	
Cancellous bone	100	0.2	
Posterior element	3500	0.25	
Sacrum	5000	0.2	
Facet	11	0.2	
*Disc*			
Endplate	24	0.4	
Nucleus pulpous	1	0.49	
Annulus ground substance	4	0.4	
Annulus fibres	360–550		0.15
*Ligaments*			
ALL	7.8		63.7
PLL	10		20
LF	15		40
CL	7.5		30
ISL	10		40
SSL	8		30
ITL	10		1.8
*Implants*			
Cage	3600	0.25	
Screws and rods	110,000	0.28	

*ALL* Anterior longitudinal ligament; *PLL* posterior longitudinal ligament; *LF* ligamentum flavum; *CL* capsular ligament; *ISL* interspinous ligament; *SSL* supraspinal ligament; *ITL* intertransverse ligament

### Model simulation

**Fig. 2:**
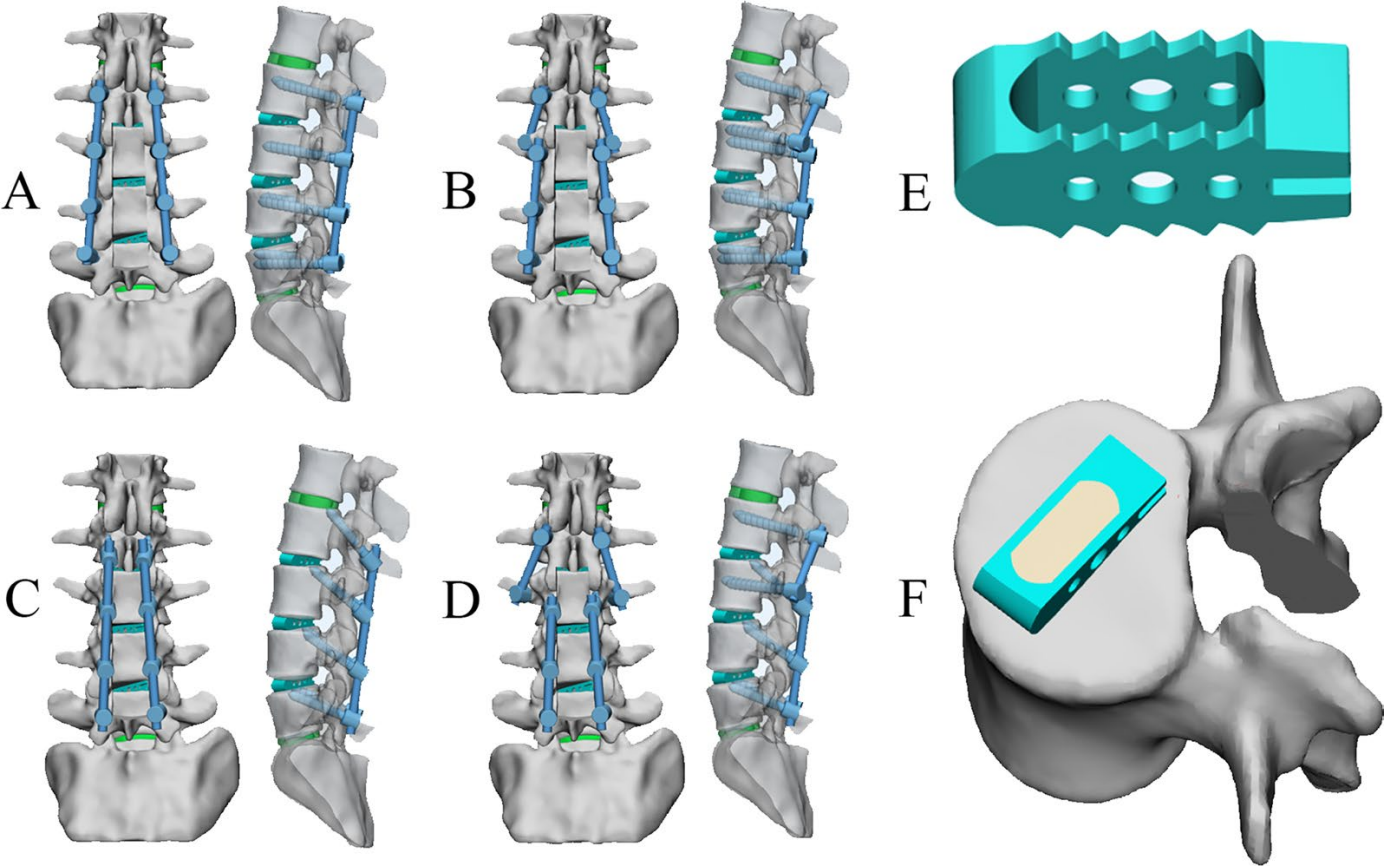
Four instrumentation constructs with different configurations for ASDz revision surgery. **A** Original internal fixation removed and traditional trajectory (TT) screws extended. **B** Original TT screws retained and dual-trajectory screws extended. **C** Original internal fixation removed and CBT screws extended. **D** Original CBT screws retained and dual-trajectory screws extended. **E** Configuration of the designed cage. **F** Bone graft and cage had successful interbody fusion

As shown in Fig. 2, four different fixation methods for ASDz revision surgery were constructed in this study. To simulate the initial posterior lumbar interbody fusion (operation), the posterior partial lamina of L3-5, the medial bone of the L3-5 adjacent segment facet joint and the intervertebral disc of L3/4 and L4/5 were removed and implanted into the fusion cage. Models A and B were implanted with TT screws. Taking the intersection of the horizontal line of the midpoint of the transverse process and the vertical line of the outer edge of the superior articular process as the insertion point, the trajectory of the screw was along the axis of the pedicle of the vertebral arch, from the outside to the inside, at an angle of 10–15 degrees with the sagittal plane. Models C and D were implanted with CBT screws according to the method by Santoni [26]. Taking the intersection of the 1 mm of the inferior margin of the transverse process and the midline of the superior articular process as the insertion point, the screws were placed from 5 o’clock to 11–12 o'clock on the left side of the pedicle isthmus, and from 7 o’clock to 12–1 o’clock on the right side, with a head inclination angle of about 25 degrees. The overlapping part between the cage and the vertebra was removed by a Boolean operation, and the cage–endplate interface was assigned a friction coefficient of 0.2 to simplify the influence of the teeth of the cage [27]. Previous studies have shown that there is a greater risk of ASDz in the proximal adjacent segment [28], so to simulate ASDz revision surgery, the partial lamina of the L2 and L2/3 intervertebral discs were removed and implanted into the cage. In models A and C, the original internal fixation was removed and the L3-5 screw rods were elongated screw rods and extended up to L2. In models B and D, the original internal fixation was retained, the second pedicle screw was placed in the L3 vertebra and extended upwards, and the partial lamina and the medial bone of the facet joint and the intervertebral disc of L2/3 were removed. The screw–rod system and cage were constructed Pro/Engineer 5.0 software, in which the diameter of the TT screw was 6 mm, the length was 45 mm, the diameter of the CBT screw was 5.5 mm, the length was 35 mm, and the size of the interbody fusion cage was 28*10*11 mm2.

### FE model validation

To validate the rationality of the model, the in vitro verification method of Renner et al. [29] was implemented, in which the bottom of the sacrum was constrained in all degrees of freedom, and the motion of the spine in the sagittal, coronal and transverse planes was defined as flexion and extension and lateral bending and axial rotation, respectively. Four pure moments (flexion: 8 N m, extension 6 N m, lateral bending ± 6 N m, rotation ± 4 N m) were applied to the centre of the upper surface of the L1 vertebra, and the ROM of each segment was measured. In addition, by applying a gradually increasing compressive preload (100–400 N) on L1, the intradiscal pressure (IDP) of L4/5 was measured and compared with the previous experimental results of Brinckmann et al. [30, 31].

### Boundary and loading conditions

The boundary conditions and loads of the FE model were loaded in ABAQUS software. In all the FE models, the bottom of the sacrum was constrained in all degrees of freedom, and a compressive preload of 400 N was applied on the upper surface of the L1 vertebra to simulate the gravity of the lumbar vertebrae under physiological conditions. A pure moment of 7.5 N m was applied to simulate flexion, extension, lateral bending and axial rotation [31].

## Results

### FE model validation

**Fig. 3:**
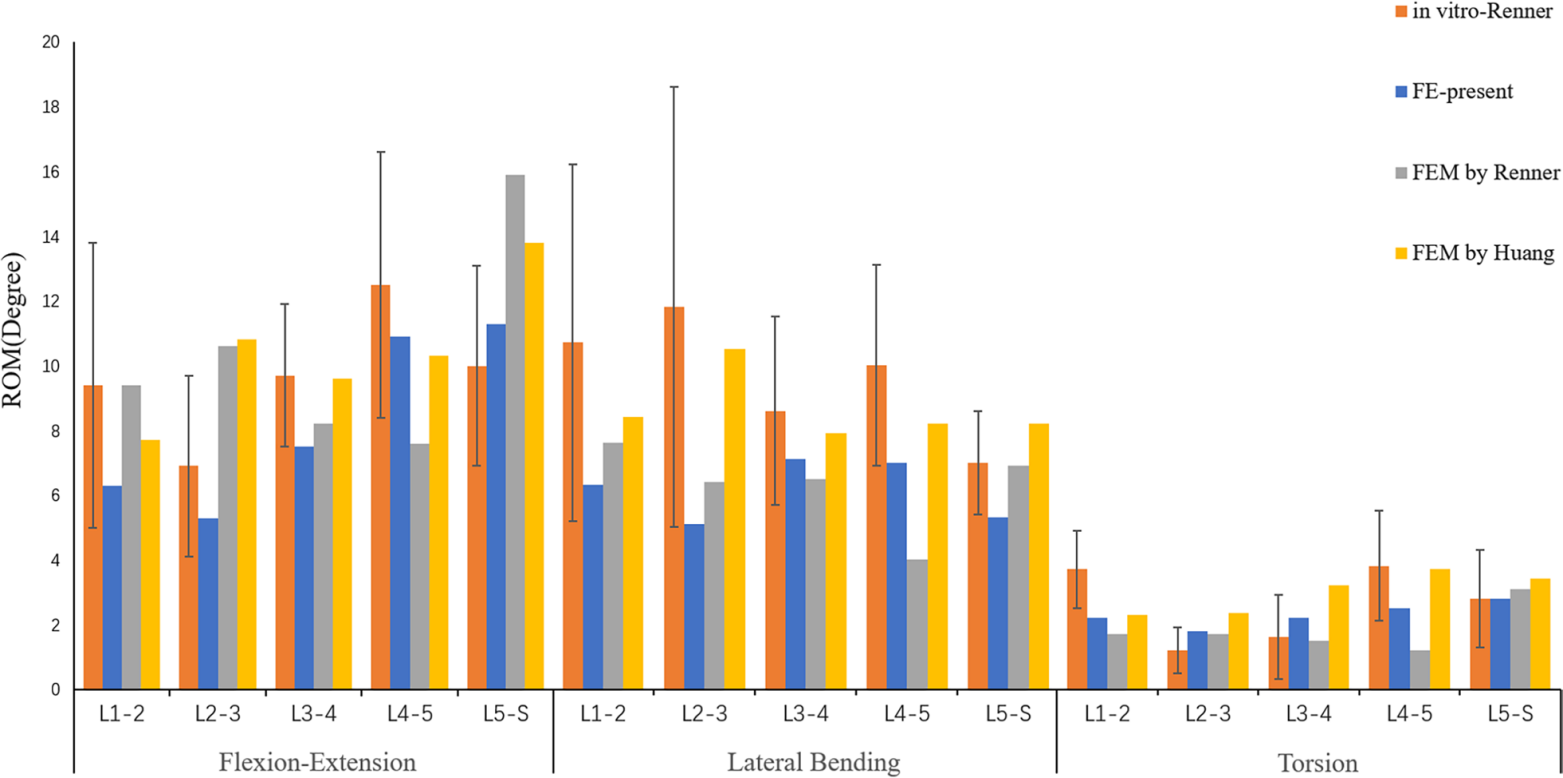
ROM of each segment (comparison with Renner et al.)

**Fig. 4:**
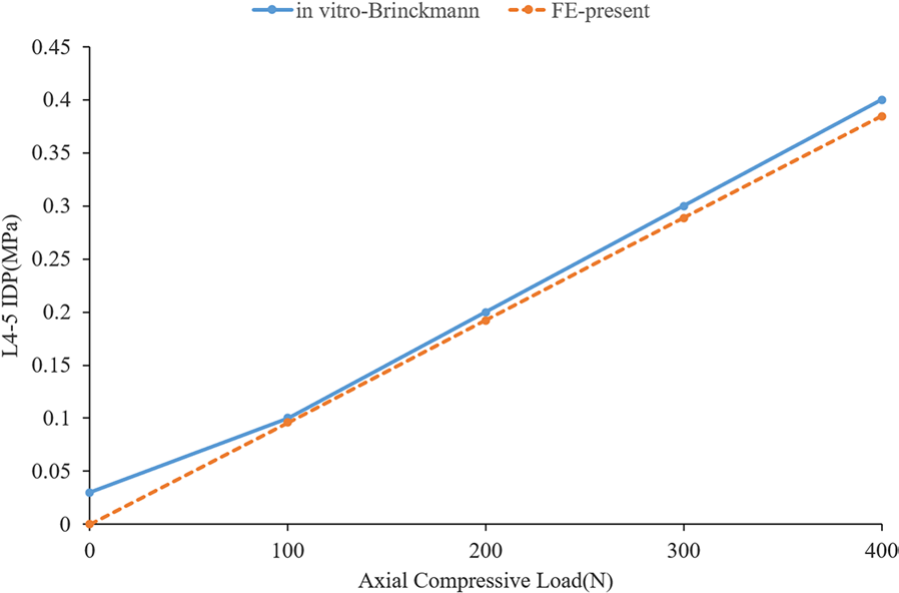
IDP of L4/5 under different compression loads (comparison with Brinckmann et al.)

In this study, the rationality of the FE model was verified using previously reported experimental methods. By applying the same loading and boundary conditions, the ROM of L1-S1 and the IDP of L4/5 were measured and compared with previous research results [25, 29, 30], as shown in Figs. 3 and 4. The ROM of each segment is in good agreement with that of previous in vitro experiments and FE studies. Under an increasing compression preload, the IDP of L4/5 also increased. Therefore, we think that the finite element models of this study are effective for the subsequent research.

### ROM of the fusion segment (L2-5)

**Fig. 5:**
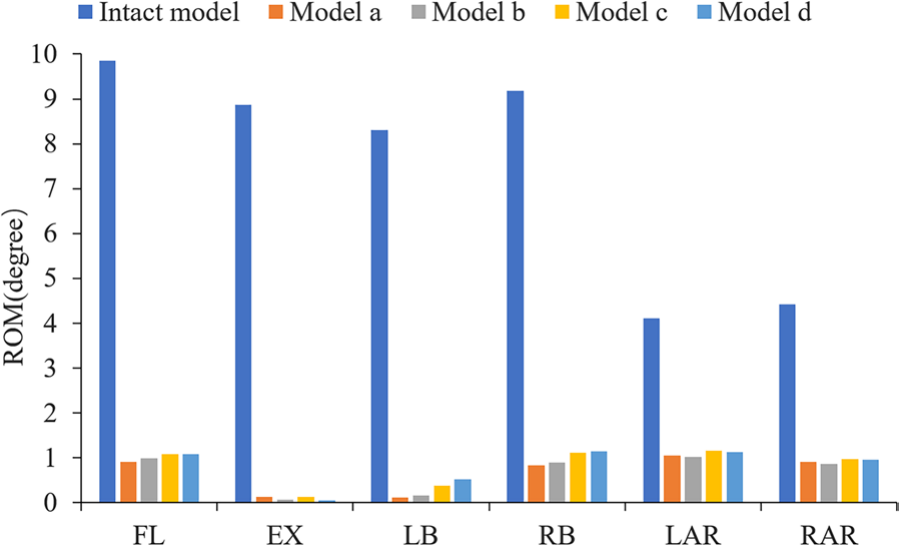
Comparison of the ROMs of different models at the fusion segment

As shown in Fig. 5, all models had the greatest restrictions on the fusion segments (L2-5) under flexion–extension conditions (89–99%) and the least restrictions under axial rotation (72–80%), with lateral bending showing a level of restriction between the two. Under all loading conditions, there was no significant difference between model A and model B, nor between model C and model D, but the restriction of the fusion segment of models A and B was greater than that of models C and D. The ROM of model C was 1.18, 1.59 and 1.09 times that of model A, in terms of flexion–extension, lateral bending and axial rotation, respectively, while the corresponding ROMs of model D were 1.07, 1.58 and 1.11 times those of model B.

### Maximum von Mises stress (VMS) of the fusion cage

**Fig. 6:**
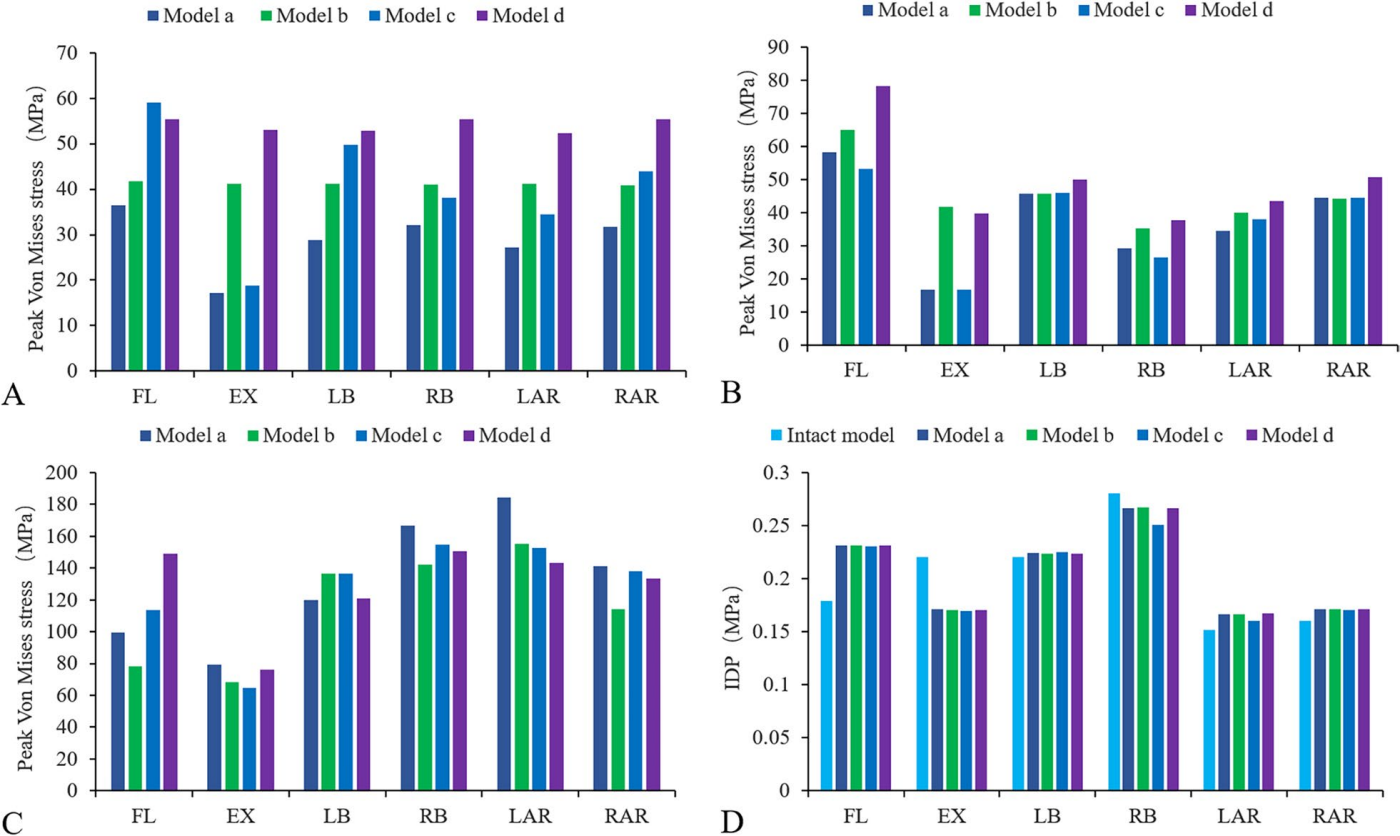
Comparison of maximum von Mises stress (MPa) between different structures of the different models. **A** Interbody cage (L2/3) for the implanted models, **B** L2/3 cage-L3 superior endplate interface, **C** internal fixation, **D** IDP of the adjacent intervertebral disc (L1/2)

The maximum von Mises stress (VMS) of the fusion cage is shown in Fig. 6A. Overall, the stress of model D was the highest, while the stress of model A was the lowest. In terms of flexion, the cage stress value of model C was the highest (59.09 MPa), but only greater by 3.7 MPa over that of model D. In terms of extension, the peak stress of model D was 3.1, 1.3 and 2.8 times that of models A, B and C, respectively. In terms of lateral bending, the stress of model C was higher than that of model B, reaching a peak of 55.47 MPa. The peak stress of the model D fusion cage was 1.8, 1.3, and 1.2 times higher than those of models A, B and C, respectively. In terms of axial rotation, the stress of model C was less than that of model B, and the peak value of model D was 1.8, 1.3 and 1.4 times that of models A, B and C, respectively.

### Peak VMS of the fusion cage–L3 superior endplate interface

**Fig. 7:**
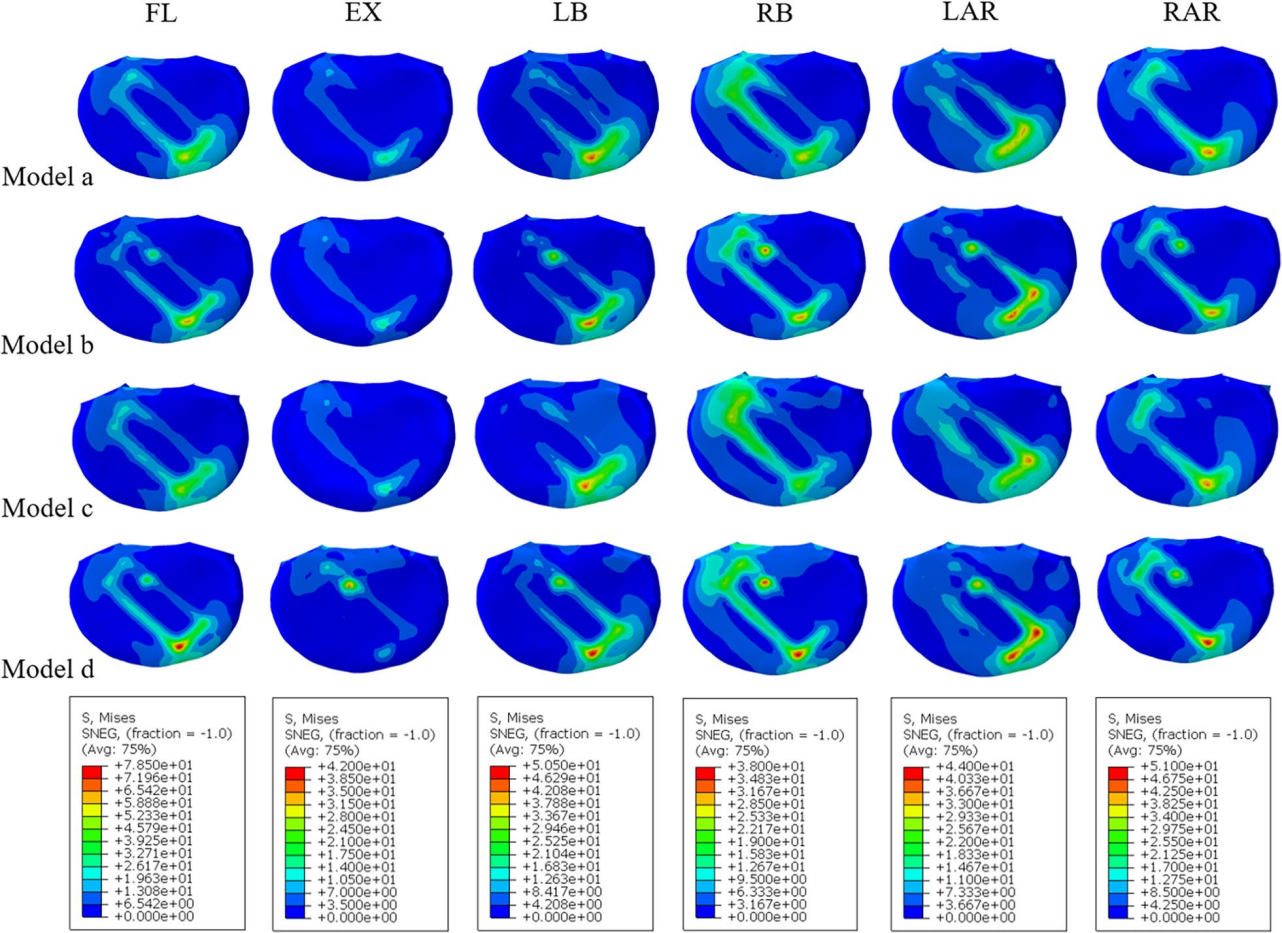
Von Mises stress (MPa) distribution of the L2/3 cage–L3 superior endplate interface for the four fixation models

As shown in Fig. 6B, except under extension, the stress value of model D was significantly higher than that of the other models, reaching the peak value during flexion (78.3 MPa). In terms of extension, the maximum peak stress was achieved by model B, but it was not substantially different from that of model D; the stresses of these two models were significantly higher than those of models A and C, respectively. In terms of lateral bending, the difference between the models is reflected in the right bending. The largest difference in stress was between model C and model D (11.38 MPa). In terms of axial rotation, the stress difference between the left axial rotation was the largest, and the maximum difference between model A and model D was 9.04 MPa. The stress distribution is shown in Fig. 7.

### VMS of the screw–rod system

**Fig. 8:**
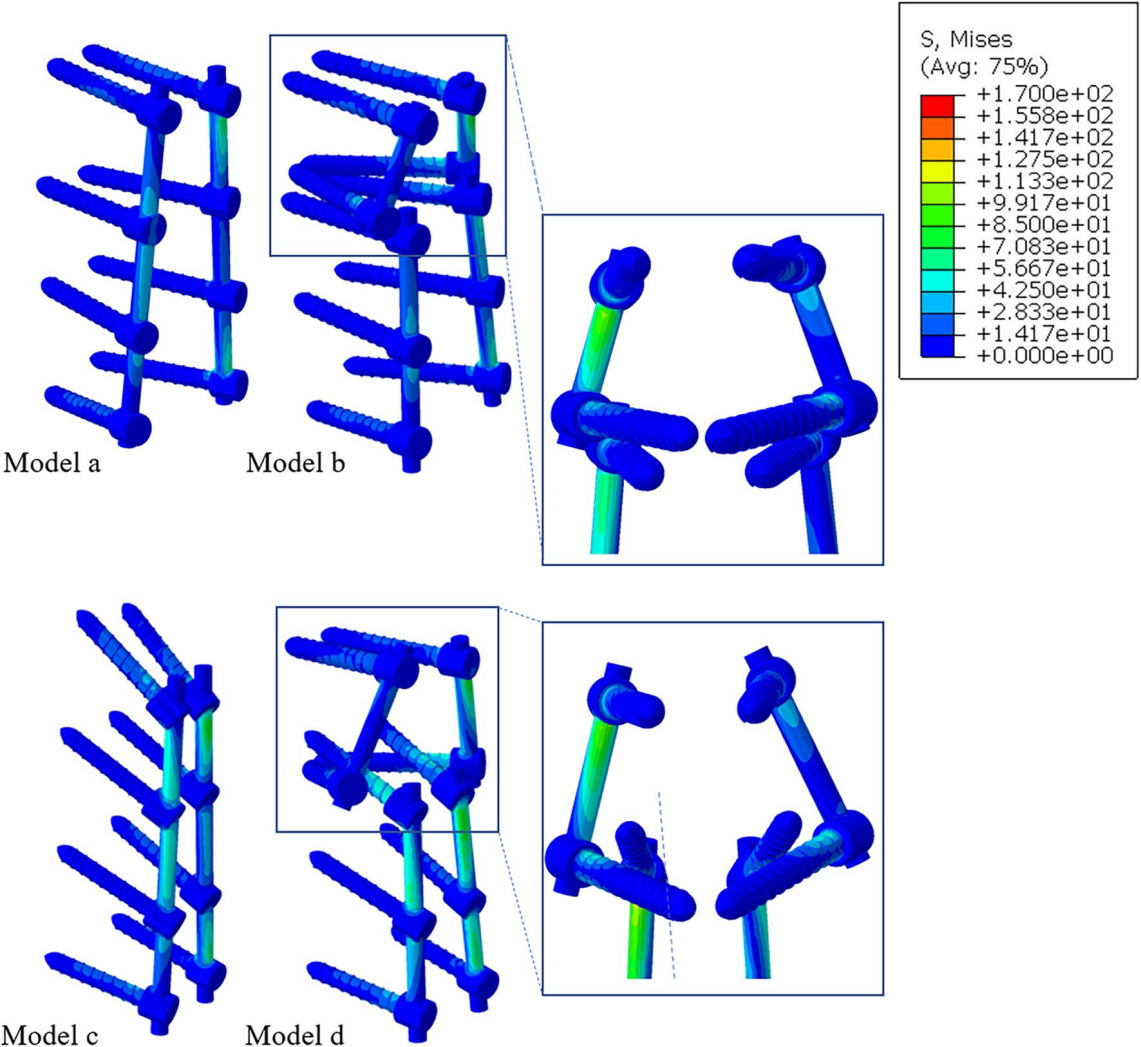
Von Mises stress (MPa) distribution of the internal fixation for the four fixation models during right bending

The study of the stress distribution of the screw rods can provide a good reference basis for loosening and fracture of the screw–rod system in the future. As shown in Fig. 6C, in terms of flexion and extension, the peak stress of model D was the highest (148.8 MPa), and the maximum difference between model D and model B was 70 MPa. For both lateral bending and axial rotation, the peak value of model A was significantly higher than that of the other models. Specifically for axial rotation, the peak difference between model A and the other models was 41 MPa, while for lateral bending, the difference was 24 MPa. In addition, the stresses of model A and model C were higher than those of model B and model D, respectively. For axial rotation, the difference between models A and B was the largest, and the difference in peak stress was 30 MPa. The difference between model C and model D was small, no greater than 10 MPa (Fig. 8).

### IDP of the adjacent intervertebral disc (L1/2)

As shown in Fig. 6D, the IDP of the L1/2 intervertebral disc was measured with the four models. Under all loading conditions, compared with the intact FE model, the IDP of the L2/3 intervertebral disc in the four models increased slightly, but nonsignificantly, in terms of left bending, left and right axial rotation, and, especially, in flexion.

## Discussion

At present, there is no unified surgical treatment plan for ASDz, but some scholars have proposed dual-trajectory screws technology. Because CBT screw not only has good advantages in biomechanics, but also has advantages that traditional pedicle screws do not have, CBT screws are more and more widely used at present. The method of combining TT screw with CBT screw can not only reduce the operation time and difficulty, but also provide good stability. However, the reoperation rate of ASDz is low; lack of long-term follow-up of patients cannot prove the superiority of this method, so the significance of this study is to use the finite element analysis to evaluate the mechanical characteristics of revision surgery for ASDz.

The purpose of this study was to analyse the biomechanical characteristics of different internal fixation methods during ASDz revision surgery and to provide a basis for the treatment of this disease. Usually, the structural stiffness of the model is used to evaluate the resistance to motion of the fusion segment [32]. The structural stiffnesses of the four models in the experimental analysis increased similarly; flexion and extension showed the most substantial changes, followed by lateral bending and axial rotation, similar to previous biomechanical research results [33]. In the fusion segment of the model, the ROM is obviously limited compared with the intact FE model, and the limit to the model can be up to 72–99%. Under all loading conditions, the difference in the ROM of the fixed segment between the model retaining the original internal fixation and prolonging fixation with dual-trajectory screws and the model removing the original internal fixation and prolonging fixation with a long bar, less than 0.1°, was not significant. In terms of axial rotation and lateral bending, the ROM of models C and D was significantly higher than that of models A and B, which is consistent with previous biomechanical reports that found that CBT screws are superior to TT screws in terms of flexion and extension resistance and a higher anti-pullout force but worse than TT screws in terms of lateral bending and axial rotation [34]. Matsukawa et al. believed that longer screws could improve the fixation strength of axial rotation of the vertebral body [34]. The longer the length of the screws, the longer the distance it can be placed into the vertebral body, reaching the middle column or even the anterior column. A longer moment arm prevented excessive rotation and lateral bending of the vertebral body. Because the biomechanical assessment of intervertebral fusion was not possible, successful intervertebral fusion was considered according to the FDA definition of bridging trabecular bone between fusion segments, a translational activity less than 3 mm, and an ROM less than 5 degrees [35]. This experimental study proved that all the models can achieve the fusion of bone graft very well. This means that the stability of retaining the original internal fixation and prolonging fixation with dual-trajectory screws is similar to that of removing the original internal fixation and prolonging fixation with a long bar. Although the ROM of models A and B was slightly smaller than that of models C and D, a higher stiffness does not mean better results, as it may accelerate the degeneration of adjacent intervertebral discs and lead to ASDz. Previous studies have also shown that the use of CBT screws alone can reduce the incidence of ASDz [36].

The results of this study showed that the peak stress of screw–rod system was concentrated at the interface between screws and rods, whether it was TT screws or CBT screws, which was consistent with the previous report by Liu et al. [37]. The peak stress of screw–rod system was the highest in axial rotation (184.3 MPa), the second highest in lateral bending (166.4 MPa), and the lowest in flexion–extension (148.8 MPa), which is consistent with previous research results. There was a significant difference in the peak stress between the four models, and the peak stress of the model retaining the original internal fixation and prolonging fixation with dual-trajectory screws was lower than that of the model removing the original internal fixation and prolonging fixation with a long bar, which may be due to the use of more screws to increase stiffness and avoid concentrating stress and screw–rod fracture, which is consistent with previous studies showing that the application of multiple screw rods reduced implant fracture and graft failure [15, 38]. The trend of the screw–rod stress in this experiment was not consistent with the ROM of the above fusion segments. The possible reason for our analysis was that, as mentioned above, the dual-trajectory screws technology used more internal fixation devices, so that the stress can be dispersed on screw–rod systems, so that the stress that should be concentrated on a certain screw rod was reduced. The maximum stress observed in this experiment was concentrated at the interface between screws and rods. The maximum stress may be on the screws or on the rods, and no matter where the maximum stress was located, if it was greater than the bearing strength of the screw–rod system, it will also cause the screw–rod fracture. Since the typical mechanical properties of titanium alloys include an ultimate bearing strength of 1380–2070 MPa and a yield strength of 825–895 MPa, the peak stress of the screw rods is within the range of their strength, but this does not mean that there is no future risk of screw loosening and fracture.

In the segment undergoing ASDz revision surgery (L2-3), the endplate stress and fusion cage stress of the different models changed similarly in all loading conditions, the stress when retaining the original internal fixation and prolonging fixation with dual-trajectory screws was greater than that when removing the original internal fixation and prolonging fixation with a long bar, and the stress of the CBT fusion cage was greater than that of TT, consistent with previous biomechanical reports [32, 39]. This corresponds to the above ROM of fusion segment; the smaller the restriction of the internal fixation system on the model, the more force the cage will bear. For the CBT screws, because of its short track length and insufficient capacity to bear the anterior column of the vertebral body, more force is concentrated on the interbody fusion cage. Although the endplate stress of the model retaining the original internal fixation and prolonging fixation with dual-trajectory screws was greater, previous reports have stated that the cortical bone failure strength ranges from 90 to 200 MPa [40, 41]. In our study, under flexion, the maximum stress of the model was 78.3 MPa, lower than the lower endpoint of 90 MPa for failure strength. Except for extension and right lateral bending, the IDP of each model in the adjacent segments was larger than that of the intact model. For long segment decompression and fixation of the spine, the degeneration of adjacent segments was accelerated, and the occurrence of ASDz was accelerated. Although the IDP of adjacent segments of each surgical model increased in general, the difference between them was not significant. The results show that different internal fixation methods did not cause significant differences in the IDP of the superior adjacent intervertebral disc.

Although the above-mentioned dual-trajectory screws might be meaningful for clinical practice, this study has some limitations. First, the finite element model data in this study are based on a 24-year-old adult male and was not statistically analysed, which is a common drawback of finite element analysis. Second, this study simplifies the FE model and uses linear materials for analysis, which does not more accurately reflect the biomechanical changes in lumbar structure. Finally, this study only involves the biomechanical characteristics of the normal bone population, but lumbar fusion is more likely to be performed for patients with osteoporosis, so it is necessary to study FE models generated from data from this population.

## Conclusions

This study shows that when the original internal fixation is retained and fixation is prolonged with dual-trajectory screws, a similar structural stability can be achieved as when the original internal fixation is removed and fixation is prolonged with a long bar. Although the former will lead to an increase in the stress of the fixed segment endplate, it is always within the destructive strength of the cortical bone and will not lead to a significant change in the IDP of the superior adjacent intervertebral disc and further reduces the risk of screw–rod fractures, regardless of whether the first operation uses CBT or TT screws. Dual-trajectory screws are thus another choice for lumbar revision surgery for ASDz.

## Data Availability

Please contact the corresponding author for data requests.

## Abbreviations

FE: Finite element
ROM: Ranges of motion
CBT: Cortical bone trajectory
TT: Traditional trajectory
PLIF: Posterior lumbar interbody fusion
IDP: Intradiscal pressure
ASDeg: Adjacent segment degeneration
ASDz: Adjacent segment disease
ALL: Anterior longitudinal ligament
PLL: Posterior longitudinal ligament
LF: Ligamentum flavum
CL: Capsular ligament
ISL: Interspinous ligament
SSL: Supraspinal ligament
ITL: Intertransverse ligament
FL: Flexion
EX: Extension
LAR: Left axial rotation
RAR: Right axial rotation
LB: Left bending
RB: Right bending
